# Time trends of socioeconomic inequalities in adolescent smoking in Okinawa, Japan, 2008–2016: a repeated cross-sectional study

**DOI:** 10.1186/s12199-021-00948-y

**Published:** 2021-02-17

**Authors:** Minoru Takakura, Masaya Miyagi, Akira Kyan

**Affiliations:** 1 School of Health sciences, Faculty of Medicine, University of the Ryukyus, 207 Uehara, Nishihara, Okinawa, 903-0215 Japan; 2 Faculty of Education, University of the Ryukyus, 1 Senbaru, Nishihara, Okinawa, 903-0213 Japan; 3 Department of Childhood Education, Okinawa Women’s Junior College, 1 Agarihama, Yonabarucho, Okinawa, 901-1304 Japan

**Keywords:** Adolescents, Cigarette use, Socioeconomic inequality, Trends, Japan

## Abstract

**Background:**

Smoking among Japanese adolescents has decreased noticeably. However, little is known whether the decreasing trend in adolescent smoking can be seen across all socioeconomic status (SES) groups. This study aimed to examine trends in socioeconomic inequalities in smoking among Japanese adolescents between 2008 and 2016.

**Methods:**

We conducted a repeated cross-sectional study using data from three surveys of high school students in Okinawa, Japan, in 2008, 2012, and 2016. The study participants consisted of 7902 students in grades 10 through 12 (15–18 years). Smoking was assessed as current cigarette use. SES indicators included familial SES (parental education and family structure) and student’s own SES (school type). To evaluate absolute and relative inequalities, prevalence differences (PDs) and ratios (PRs) between low and high SES groups were estimated. The slope index of inequality (SII) and relative index of inequality (RII) were also calculated.

**Results:**

Smoking prevalence among boys and girls significantly declined from 11.5% and 6.2% in 2008 to 4.7% and 1.9% in 2016, respectively. Similar decreasing trends in smoking were found among most of the SES groups. The PDs and SII for parental education in boys and family structure in girls decreased over time while those for school type persisted among boys and girls. The PRs and RII for school type in boys increased while those for other SES indicators among both sexes remained stable over time.

**Conclusions:**

Smoking among Japanese adolescents has been declining and time trends of socioeconomic inequalities in smoking varied by absolute and relative measures. Further policies and/or interventions to reduce smoking inequalities should focus on the context of schools, especially in vocational high schools.

## Background

Smoking is a leading preventable cause of early death and disability worldwide [1]. In Japan, smoking is also the top risk factor contributing to mortality from non-communicable diseases [2]. As smoking mostly begins during adolescence, smoking prevention among adolescents is critical to decreasing mortality and morbidity [3]. To develop effective smoking prevention programs, monitoring time trends in smoking and understanding its determinants are needed.

In recent decades, time trends in smoking prevalence among young people have shown a steady decline at the global level [4]. The cross-national studies, such as the European School Survey Project on Alcohol and Other Drugs and the Health Behaviour in School-aged Children study have shown that a general decreasing trend in cigarette smoking was observed in many European countries [5, 6]. The prevalence of smoking among Japanese adolescents was also trending downward over time [7, 8]. Nationwide surveys of high school students showed that current smoking prevalence among boys was 30.7% in 1996 and 3.5% in 2014, whereas among girls it was 12.6% and 1.4%, respectively [7].

Although adolescent smoking prevalence is declining, this progress is not even across population subgroups [4]. For example, those with low socioeconomic status (SES) were more likely to smoke than those with high SES [9], but previous research has shown socioeconomic inequalities in adolescent smoking trends [10–14]. Socioeconomic inequalities are often assessed by both absolute measures, such as prevalence differences and slope index of inequality, and relative measures, such as prevalence ratios, odds ratios, and relative index of inequality [15]. A study conducted in seven European countries that used educational tracks as the SES indicator found that trends in absolute educational differences in adolescent smoking (i.e., prevalence differences between vocational students and academic students) varied across the countries while relative educational differences (i.e., odd ratios for vocational students compared with academic students) had a tendency to increase in all countries between 2002 and 2010 [12]. A Danish study which assessed parents’ occupational social class found that the absolute socioeconomic differences in daily smoking among 15-year-olds increased from 1991 to 2006 and declined from 2006 to 2014 and the relative socioeconomic differences increased over 1991–2014 [10]. A Finnish study created a cumulative socioeconomic adversity variable which combined parental education, parental unemployment, and family structure and showed that the overall prevalence of adolescent smoking decreased between 2000 and 2015. However, no similar changes were observed among adolescents with most socioeconomic adversities, which resulted in the increased relative socioeconomic differences over time [11]. On the other hand, a German study which assessed parental SES (family affluence) and student’s own SES (school type) found that both socioeconomic differences in adolescent smoking remained unchanged between 1994 and 2002 [13]. In a Slovak study which measured parental education, the socioeconomic differences in adolescent smoking decreased among boys and increased among girls between 1998 and 2006 [14].

In Japan, socioeconomic differences in adolescent smoking were also shown, indicating that those with low parental education, not living with both parents, or attending vocational high schools were more likely to smoke [16]. However, no study has reported time trends in socioeconomic differences in smoking among Japanese adolescents so far. This is surprising because income inequality in Japan has increased over the past several decades [17] and Japan’s child poverty rate was 13.9 % in 2015, which was higher than the average across the Organization for Economic Co-operation and Development countries [18]. A study on socioeconomic trends among Japanese adults showed that smoking inequalities in both absolute and relative measures widened between 2000 and 2010, especially among women [19]. Yet, it is not clear whether the findings of adult populations in Japan can be true of adolescent populations.

Measurements of SES in adolescents are categorized into two dimensions: familial SES and adolescent’s own SES [13, 20]. Parental education and family structure are familial SES indicators and school type reflects the adolescent’s own SES [13, 20]. Each SES indicator was associated with adolescent smoking in previous studies [9, 11–14, 20]. The poverty rate in two-adult households with at least one child in Japan was 10.7%, while that in single-adult households with at least one child was 50.8% in 2015 [18, 21]. Therefore, family structure is a suitable indicator of SES. In Japan, after completing compulsory education, nearly all students go on to upper secondary schools known as senior high schools. Of them, roughly 70% attend general high schools and 30% attend vocational and specialized high schools [22]. As the advancement rates to tertiary education in general and vocational high schools in 2016 were 64.1% and 20.9%, respectively [22], school type can predict the future education level.

In sum, previous findings on socioeconomic trends in adolescent smoking were inconsistent and suggested that socioeconomic trends in adolescent smoking may depend upon study population, survey years, and the type of the SES indicators. In this study, we analyzed time trends in socioeconomic differences in smoking among Japanese high school students between 2008 and 2016. We included parental education, family structure, and school type as the SES indicators and assessed both absolute and relative differences of these indicators.

## Methods

### Study design and setting

We conducted a repeated cross-sectional study using data from three prefecture-wide representative surveys of public high schools in Okinawa Prefecture, Japan, in 2008, 2012, and 2016. Okinawa Prefecture, which is located in the southwesternmost part of Japan, has a population of 1.4 million. Okinawa Prefecture had about 60 public high schools during the survey period and is divided into six regions (four are situated on Okinawa Island while two are in remote islands). The surveys were designed to obtain comprehensive data on health, sociodemographic, and psychosocial information and to explore those trends among high school students across the prefecture [8].

### Sampling and data collection

For all surveys, we employed the same sampling strategy. Schools included in the study were randomly selected one or more from each school type with a probability that was proportional to the number of schools within school types and regions in the prefecture. In each school, one class was chosen from each grade. Each survey used a similar data collection procedure. After permission to conduct the study was obtained from the principals of the study schools, classroom teachers distributed an anonymous self-administered questionnaire in classrooms from September to December. After providing information about the purpose and the ethical considerations of the study, all students attending the class were requested to complete and return the questionnaire sealed in an unmarked envelope. Students were free to decline to participate in the study at any time during the survey. The students were informed prior to administering the survey that returning the questionnaire constituted informed consent. No follow-up was conducted on students absent from school when the survey was conducted.

### Participants

During the survey periods, we recruited a total of 10,075 students (3248 in 2008; 3386 in 2012; 3441 in 2016) enrolled in grades 10 through 12 (aged 15–18 years) in the study schools (29 schools in 2008 and 30 schools in 2012 and 2016). Among them, 8955 students participated in the surveys (513 declined to participate and 607 were absent from school). Of these, we excluded 1053 students with missing variables of interest. Finally, complete data on all variables of interest were available for 7902 students, which was 78.4% of the original sample (2528 in 2008; 2651 in 2012; 2723 in 2016), and these data were used for analysis.

The study was conducted in accordance with the Declaration of Helsinki, and the study protocols were approved by the Institutional Review Board of the University of the Ryukyus (No. 21, 139, 343).

### Measures

Smoking was assessed using a question adapted from the Youth Risk Behavior Surveillance conducted by the US Centers for Disease Control and Prevention [23]. We evaluated current cigarette use by the following question: “During the past 30 days, on how many days did you smoke cigarettes?” Respondents who smoked cigarettes on at least 1 day in the past month were classified as current smokers [24]. Test-retest reliability of this question demonstrated moderate stability for Japanese adolescents, with kappa statistics of 0.51 [25].

The SES indicators included parental education, family structure, and school type. Parental education was based on information gathered from students about their mother’s or father’s educational attainment. The higher level of education attained by either parent was included in the analysis. The categories used for analysis were low (high school or less), middle (specialized training college or junior college), and high (university or more). These categories are consistent with the International Standard Classification of Education levels, 1/2/3, 5, and 6 or more, respectively [26]. Family structure was based on the person with whom the student was living. The response was categorized into three groups: “living with both parents,” “single parent,” and “others.” It was not clear whether their parent was a biological parent or a stepparent. School type was categorized into general high schools and vocational high schools.

### Statistical analyses

All analyses were stratified by sex, as previous studies have shown clear differences in adolescent smoking between boys and girls [7, 8]. Initially, the prevalence and 95% confidence interval (CI) of smoking was estimated by each SES indicator and each survey year. Cochran-Armitage test for trend was used to assess time trends in smoking prevalence over time by each SES category. Chi-square test was performed to evaluate differences in smoking prevalence among different categories for each SES indicator by each survey year. When the sample size was small, Fisher’s exact test was conducted. Next, socioeconomic inequalities in smoking between low and high SES groups were assessed by absolute and relative measures. Both absolute and relative measures were estimated with 95% CIs for each SES indicator and each survey year. For the absolute measures, prevalence differences (PDs) [10, 12, 15, 27] and the slope index of inequality (SII) [28–30] were calculated using generalized linear models with binomial distribution and identify link function. The coefficient yields an estimate of the absolute inequality. When this binomial model failed to converge, a generalized linear model with normal distribution and identify link function was used [31]. For the relative measures, prevalence ratios (PRs) [15] and the relative index of inequality (RII) [28–30] were calculated using generalized linear models with binomial distribution and log link function. The exponentiated coefficient yields an estimate of the relative inequality. The SII and RII were estimated using ridit score for each SES indicator as an independent variable. The PDs and PRs are simple measures of inequality, which are pairwise comparisons of smoking prevalence between low and high SES groups [27]. The SII and RII are summary measures of inequality as the changes in smoking between the bottom and top points in the SES hierarchy while accounting for the cumulative distribution in each SES [27]. Time trends of absolute and relative measures were assessed by the inclusion of the interaction term between each SES indicator (ridit score for the SII and RII) and survey year [27, 28]. In the model, survey year was treated as a continuous variable coded as 1 for 2008, 2 for 2012, and 3 for 2016 [28] and Wald test was used to test if the interaction term was statistically significant. Finally, these models were adjusted for sociodemographic factors, such as grade and region, which were considered as potential confounders.

## Results

Table 1 shows the distribution of study participants by sociodemographic characteristics and survey years. Chi-square tests indicate significant relationships between survey year and region, parental education, and family structure (*P* < 0.05).

**Table 1:** Distribution of study participants by sociodemographic characteristics

Survey year										
		All years		2008		2012		2016		
		*N*	(%)	*N*	(%)	*N*	(%)	*N*	(%)	*P* ^a^
**Total**		7902	(100)	2528	(100)	2651	(100)	2723	(100)	
Gender	Boys	3742	(47.4)	1230	(48.7)	1218	(45.9)	1294	(47.5)	0.145
	Girls	4160	(52.6)	1298	(51.3)	1433	(54.1)	1429	(52.5)	
Grade	10th	2662	(33.7)	873	(34.5)	914	(34.5)	875	(32.1)	0.212
	11th	2650	(33.5)	856	(33.9)	873	(32.9)	921	(33.8)	
	12th	2590	(32.8)	799	(31.6)	864	(32.6)	927	(34.0)	
Region	Okinawa Island	7005	(88.6)	2183	(86.4)	2370	(89.4)	2452	(90.0)	<0.001
	Remote islands	897	(11.4)	345	(13.6)	281	(10.6)	271	(10.0)	
Parental education	High	2579	(32.6)	772	(30.5)	962	(36.3)	845	(31.0)	<0.001
	Middle	1937	(24.5)	613	(24.3)	595	(22.4)	729	(26.8)	
	Low	3386	(42.9)	1143	(45.2)	1094	(41.3)	1149	(42.2)	
Family structure	Both parents	5670	(71.8)	1840	(72.8)	1911	(72.1)	1919	(70.5)	0.038
	Others	395	(5.0)	128	(5.1)	146	(5.5)	121	(4.4)	
	Single parent	1837	(23.2)	560	(22.2)	594	(22.4)	683	(25.1)	
School type	General HS	5391	(68.2)	1701	(67.3)	1839	(69.4)	1851	(68.0)	0.258
	Vocational HS	2511	(31.8)	827	(32.7)	812	(30.6)	872	(32.0)	

*HS* high school

^a^Chi-square test

Table 2 shows time trends in smoking prevalence by each SES indicator stratified by sex. Overall, smoking prevalence among boys and girls significantly declined from 11.5% and 6.2% in 2008 to 4.7% and 1.9% in 2016, respectively. The declining trends in smoking were observed for most of SES groups. However, there were no significant trends among boys with the highest parental education (*P* for trend = 0.183) and girls belonging to the “others” group for family structure (*P* for trend = 0.111). The prevalence in these groups remained constant over time. Table 2 also shows the results of chi-square tests of homogeneity. There were significant differences in smoking prevalence among most of the SES categories, indicating that smoking was more prevalent among low SES categories than high SES categories. However, parental education categories among boys in 2016 and family structure categories among boys in 2012 and both sexes in 2016 did not show any significant differences in smoking prevalence.

**Table 2:** Time trends in the prevalence of current smoking between 2008 and 2016 according to the SES indicators by sex

	2008					2012					2016					
			Current smoking					Current smoking					Current smoking			
	*N*	(%)	*n*	%	(95% CI)	*N*	(%)	*n*	%	(95% CI)	*N*	(%)	*n*	%	(95% CI)	*P* for trend ^a^
**Boys**	1230	(100)	141	11.5	(9.7, 13.2)	1218	(100)	91	7.5	(6.0, 8.9)	1294	(100)	61	4.7	(3.6, 5.9)	<0.001
**Parental education**																
High	396	(32.2)	25	6.3	(3.9, 8.7)	463	(38.0)	31	6.7	(4.4, 9.0)	448	(34.7)	19	4.2	(2.4, 6.1)	0.183
Middle	262	(21.3)	31	11.8	(7.9, 15.7)	240	(19.7)	11	4.6	(1.9, 7.2)	315	(24.3)	11	3.5	(1.5, 5.5)	<0.001
Low	572	(46.5)	85	14.9	(11.9, 17.8)	515	(42.3)	49	9.5	(7.0, 12.0)	531	(41.0)	31	5.8	(3.8, 7.8)	<0.001
*P* for chi-square test				<0.001					0.041					0.251		
**Family structure**																
Both parents	907	(73.7)	88	9.7	(7.8, 11.6)	870	(71.4)	56	6.4	(4.8, 8.1)	941	(72.7)	40	4.3	(3.0, 5.5)	<0.001
Others	62	(5.0)	11	17.7	(8.2, 27.3)	79	(6.5)	6	7.6	(1.8, 13.4)	55	(4.3)	2	3.6	(-1.3, 8.6)	0.009
Single parent	261	(21.2)	42	16.1	(11.6, 20.5)	269	(22.1)	29	10.8	(7.1, 14.5)	298	(23.0)	19	6.4	(3.6, 9.1)	<0.001
*P* for chi-square test				0.005					0.065^b^					0.345^b^		
**School type**																
General HS	809	(65.8)	79	9.8	(7.7, 11.8)	777	(63.8)	34	4.4	(2.9, 5.8)	889	(68.7)	21	2.4	(1.4, 3.4)	<0.001
Vocational HS	421	(34.2)	62	14.7	(11.3, 18.1)	441	(36.2)	57	12.9	(9.8, 16.1)	405	(31.3)	40	9.9	(7.0, 12.8)	0.036
*P* for chi-square test				0.010					<0.001					<0.001		
**Girls**	1298	(100)	80	6.2	(4.9, 7.5)	1433	(100)	40	2.8	(1.9, 3.6)	1429	(100)	27	1.9	(1.2, 2.6)	<0.001
**Parental education**																
High	376	(29.0)	17	4.5	(2.4, 6.6)	499	(34.8)	9	1.8	(0.6, 3.0)	397	(27.8)	3	0.8	(-0.1, 1.6)	<0.001
Middle	351	(27.0)	14	4.0	(1.9, 6.0)	355	(24.8)	5	1.4	(0.2, 2.6)	414	(29.0)	6	1.4	(0.3, 2.6)	0.021
Low	571	(44.0)	49	8.6	(6.3, 10.9)	579	(40.4)	26	4.5	(2.8, 6.2)	618	(43.2)	18	2.9	(1.6, 4.2)	<0.001
*P* for chi-square test				0.006					0.008^b^					0.040^b^		
**Family structure**																
Both parents	933	(71.9)	42	4.5	(3.2, 5.8)	1041	(72.6)	17	1.6	(0.9, 2.4)	978	(68.4)	14	1.4	(0.7, 2.2)	<0.001
Others	66	(5.1)	6	9.1	(2.2, 16.0)	67	(4.7)	2	3.0	(-1.1, 7.1)	66	(4.6)	2	3.0	(-1.1, 7.2)	0.111
Single parent	299	(23.0)	32	10.7	(7.2, 14.2)	325	(22.7)	21	6.5	(3.8, 9.1)	385	(26.9)	11	2.9	(1.2, 4.5)	<0.001
*P* for chi-square test				<0.001^b^					<0.001^b^					0.123^b^		
**School type**																
General HS	892	(68.7)	46	5.2	(3.7, 6.6)	1062	(74.1)	21	2.0	(1.1, 2.8)	962	(67.3)	10	1.0	(0.4, 1.7)	<0.001
Vocational HS	406	(31.3)	34	8.4	(5.7, 11.1)	371	(25.9)	19	5.1	(2.9, 7.4)	467	(32.7)	17	3.6	(1.9, 5.3)	0.003
*P* for chi-square test				0.025					0.002					0.001		

*HS* high school, *CI* confidence interval

^a^Cochran-Armitage trend test

^b^Fisher’s exact test

Table 3 shows time trends of the PDs and PRs in smoking for the SES indicators. The PDs for parental education in boys decreased from 8.5% (95% CI 4.8–12.3) in 2008 to 1.6% (95% CI −1.1 to 4.3) in 2016 (*P* for trend = 0.018). The PDs for family structure in girls also decreased from 6.2% (95% CI 2.5–9.9) in 2008 to 1.4% (95% CI −0.4 to 3.2) in 2016 (*P* for trend = 0.025). In addition, there were no significant absolute differences for parental education among boys in 2012 and 2016 and for family structure among both sexes in 2016. On the other hand, the PRs for school type in boys increased from 1.51 (95% CI 1.11–2.06) in 2008 to 4.18 (95% CI 2.50–7.00) in 2016 (*P* for trend < 0.001). Similarly, the PRs for school type in girls increased from 1.62 (95% CI 1.06–2.49) in 2008 to 3.50 (95% CI 1.62–7.59), but this trend was not statistically significant (*P* for trend = 0.053). For other SES indicators, the PDs and PRs in smoking among both sexes persisted over time. The adjusted models showed almost the same results as the crude models.

**Table 3:** Prevalence differences and ratios in current smoking according to the SES indicators by survey year and sex

	2008		2012		2016		***P*** for trend ^c^
**Boys**							
**Parental education**							
Crude PD (95%CI)	8.5	(4.8, 12.3)	2.8	(−0.6, 6.2)	1.6	(−1.1, 4.3)	0.018
Adjusted PD (95%CI)^a^	8.9	(5.2, 12.6)	2.7	(−0.8, 6.1)	2.1	(−0.4, 4.6)	0.016
Crude PR (95%CI)	2.35	(1.54, 3.61)	1.42	(0.92, 2.19)	1.38	(0.79, 2.40)	0.082
Adjusted PR (95%CI)^a^	2.38	(1.55, 3.64)	1.39	(0.90, 2.14)	1.38	(0.79, 2.41)	0.084
**Family structure**							
Crude PD (95%CI)	6.4	(1.5, 11.2)	4.3	(0.3, 8.4)	2.1	(−0.9, 5.2)	0.117
Adjusted PD (95%CI)^a^	6.4	(1.6, 11.3)	4.4	(0.3, 8.4)	1.9	(−0.9, 4.8)	0.132^b^
Crude PR (95%CI)	1.66	(1.18, 2.33)	1.67	(1.09, 2.57)	1.50	(0.88, 2.55)	0.494
Adjusted PR (95%CI)^a^	1.65	(1.17, 2.32)	1.69	(1.11, 2.59)	1.54	(0.91, 2.61)	0.459
**School type**							
Crude PD (95%CI)	5.0	(1.0, 8.9)	8.5	(5.1, 12.0)	7.5	(4.4, 10.6)	0.467
Adjusted PD (95%CI)^a^	5.3	(1.2, 9.4)	8.6	(5.2, 12.0)	7.4	(4.3, 10.5)^b^	0.369^b^
Crude PR (95%CI)	1.51	(1.11, 2.06)	2.95	(1.96, 4.44)	4.18	(2.50, 7.00)	<0.001
Adjusted PR (95%CI)^a^	1.56	(1.14, 2.13)	2.95	(1.96, 4.43)	4.07	(2.43, 6.80)	<0.001
**Girls**							
**Parental education**							
Crude PD (95%CI)	4.1	(0.9, 7.2)	2.7	(0.6, 4.7)	2.2	(0.6, 3.7)	0.132
Adjusted PD (95%CI)^a^	3.6	(0.6, 6.7)	2.6	(0.6, 4.6)	2.2	(0.6, 3.7)^b^	0.213^b^
Crude PR (95%CI)	1.90	(1.11, 3.24)	2.49	(1.18, 5.26)	3.85	(1.14, 13.00)	0.516
Adjusted PR (95%CI)^a^	1.87	(1.09, 3.19)	2.41	(1.14, 5.10)	3.84	(1.14, 12.95)	0.510
**Family structure**							
Crude PD (95%CI)	6.2	(2.5, 9.9)	4.8	(2.0, 7.6)	1.4	(−0.4, 3.2)	0.025
Adjusted PD (95%CI)^a^	6.3	(2.6, 10.1)	4.9	(2.1, 7.7)	1.6	(−0.2, 3.3)	0.066^b^
Crude PR (95%CI)	2.38	(1.53, 3.70)	3.96	(2.11, 7.41)	2.00	(0.91, 4.36)	0.994
Adjusted PR (95%CI)^a^	2.40	(1.55, 3.73)	4.07	(2.18, 7.61)	2.07	(0.95, 4.51)	0.990
**School type**							
Crude PD (95%CI)	3.2	(0.2, 6.3)	3.1	(0.7, 5.5)	2.6	(0.8, 4.4)	0.570
Adjusted PD (95%CI)^a^	2.8	(−0.4, 5.9)	3.2	(0.8, 5.6)	2.7	(0.9, 4.5)	0.722^b^
Crude PR (95%CI)	1.62	(1.06, 2.49)	2.59	(1.41, 4.76)	3.50	(1.62, 7.59)	0.053
Adjusted PR (95%CI)^a^	1.61	(1.05, 2.46)	2.62	(1.43, 4.82)	3.44	(1.59, 7.46)	0.054

*PD* prevalence difference (%), *PR* prevalence ratio, *CI* confidence interval

^a^Adjusted for grade and region

^b^Generalized linear model with normal distribution and identify link function

^c^Wald test of the interaction term between each SES indicator and survey year

Table 4 shows time trends in the SII and RII for each SES indicator. We observed the same trends as the PDs and PRs. The SII for parental education in boys and family structure in girls decreased over time (*P* for trend = 0.005 and 0.006). The SII for family structure in boys also decreased, although it was not statistically significant (*P* for trend = 0.066). On the other hand, the RIIs for school type in boys and girls increased over time (*P* for trend < 0.001 and 0.054). The adjusted models showed the same results as the crude models.

**Table 4:** Slope index of inequality (SII) and relative index of inequality (RII) in current smoking according to the SES indicators by survey year and sex

	2008		2012		2016		***P*** for trend ^c^
**Boys**							
**Parental education**							
Crude SII (95%CI)	14.2	(7.7, 20.6)	4.5	(-0.7, 9.8)	2.5	(−1.5, 6.6)	0.005
Adjusted SII (95%CI)^a^	14.8	(8.3, 21.3)	4.6	(-1.1, 10.4)^b^	3.1	(−0.4, 6.6)	0.004
Crude RII (95%CI)	3.56	(1.89, 6.71)	1.96	(0.92, 4.17)	1.80	(0.71, 4.54)	0.180
Adjusted RII (95%CI)^a^	3.60	(1.91, 6.79)	1.87	(0.88, 3.99)	1.82	(0.72, 4.58)	0.187
**Family structure**							
Crude SII (95%CI)	13.2	(4.2, 22.1)	7.5	(0.4, 14.5)	3.6	(−2.0, 9.1)	0.066
Adjusted SII (95%CI)^a^	13.2	(4.3, 22.1)	7.5	(0.4, 14.7)^b^	3.3	(−1.8, 8.4)	0.076
Crude RII (95%CI)	2.76	(1.48, 5.16)	2.51	(1.14, 5.55)	2.04	(0.74, 5.62)	0.625
Adjusted RII (95%CI)^a^	2.76	(1.48, 5.15)	2.55	(1.16, 5.62)	2.16	(0.79, 5.94)	0.685
**School type**							
Crude SII (95%CI)	9.9	(2.0, 17.8)	17.1	(10.2, 24.0)	15.0	(8.9, 21.2)	0.449
Adjusted SII (95%CI)^a^	10.6	(2.4, 18.9)	17.1	(10.3, 24.0)^b^	14.7	(8.6, 20.9)^b^	0.362^b^
Crude RII (95%CI)	2.27	(1.22, 4.24)	8.72	(3.86, 19.74)	17.48	(6.24, 48.96)	<0.001
Adjusted RII (95%CI)^a^	2.42	(1.29, 4.54)	8.70	(3.86, 19.62)	16.54	(5.91, 46.30)	<0.001
**Girls**							
**Parental education**							
Crude SII (95%CI)	6.4	(1.9, 10.8)	3.9	(1.0, 6.7)	3.3	(1.0, 5.6)	0.170
Adjusted SII (95%CI)^a^	5.8	(1.4, 10.2)	4.3	(1.0, 7.6)^b^	3.4	(0.9, 6.0)^b^	0.210^b^
Crude RII (95%CI)	3.37	(1.44, 7.89)	5.62	(1.6, 19.75)	7.70	(1.51, 39.23)	0.314
Adjusted RII (95%CI)^a^	3.31	(1.41, 7.75)	5.23	(1.5, 18.23)	7.87	(1.54, 40.31)	0.309
**Family structure**							
Crude SII (95%CI)	11.8	(5.0, 18.6)	8.6	(3.7, 13.4)	2.9	(−0.6, 6.3)	0.006
Adjusted SII (95%CI)^a^	11.9	(5.2, 18.6)	8.9	(3.9, 13.9)^b^	3.1	(−0.2, 6.4)	0.019^b^
Crude RII (95%CI)	5.25	(2.30, 12.02)	13.38	(4.00, 44.76)	3.88	(0.89, 16.81)	0.931
Adjusted RII (95%CI)^a^	5.32	(2.33, 12.18)	14.12	(4.23, 47.14)	4.13	(0.95, 17.92)	0.899
**School type**							
Crude SII (95%CI)	6.4	(0.3, 12.6)	6.3	(1.5, 11.1)	5.2	(1.6, 8.8)	0.587
Adjusted SII (95%CI)^a^	5.6	(-0.7, 11.9)	6.4	(1.6, 11.1)^b^	5.4	(1.8, 8.9)	0.716^b^
Crude RII (95%CI)	2.64	(1.12, 6.20)	6.71	(1.98, 22.68)	12.26	(2.61, 57.58)	0.054
Adjusted RII (95%CI)^a^	2.58	(1.09, 6.07)	6.88	(2.03, 23.26)	11.85	(2.52, 55.64)	0.058

*SII* slope index of inequality (%), *RII* relative index of inequality, *CI* confidence interval

^a^Adjusted for grade and region

^b^Generalized linear model with normal distribution and identify link function

^c^Wald test of the interaction term between each SES indicator (ridit score) and survey year

## Discussion

This study showed that time trends in socioeconomic inequalities in smoking among Japanese adolescents varied by SES indicators as well as between absolute and relative measures. The absolute inequalities in smoking for parental education in boys decreased over time. This trend was due to a decreasing prevalence in the low parental education group while remaining unchanged at low levels in the high parental education group. The absolute inequalities for family structure in girls also decreased over time. In addition, the absolute differences for family structure in 2016 among both sexes disappeared, indicating no difference in smoking prevalence between students in intact families and students in non-intact families in the latest study period. Accordingly, the absolute inequalities in smoking for the familial SES indicators, such as parental education and family structure, seem to decrease from 2008 to 2016. Conversely, the relative inequalities in smoking for school type increased during the study period. As both of the simple and summary measures of inequality showed the same results, there was robust evidence for these trends in socioeconomic inequalities in smoking among Japanese adolescents.

The relative measure of socioeconomic inequality is likely to be unstable by a mathematical consequence when smoking prevalence is very low [12]. In fact, Holstein et al. [10] argued that “a high relative social inequality in smoking may not be so important if the smoking prevalence is low and/or declining rapidly.” Meanwhile, although the absolute measure may decrease when smoking prevalence decreases [30], it represents the excess number of adolescent smokers from lower SES groups [10], and thus is appropriate to evaluate the effect of public health policies to reduce socioeconomic inequalities in smoking [30]. As this study showed a low and decreasing prevalence of smoking, we gave more emphasis to the absolute measure when interpreting time trends in socioeconomic inequalities in smoking. The present finding about the narrowing in absolute inequalities in smoking for familial SES indicators among Japanese adolescents is a desirable result from the viewpoint of closing the gap. Our findings were partly consistent with a Slovak study [14] that found that the socioeconomic differences in smoking for parental education decreased among boys and increased among girls over time. However, some previous studies showed that time trends in smoking differences between familial SES groups have persisted or increased over time [10, 11, 13, 20].

A possible explanation for our findings is that recent changes in tobacco price policies in Japan might affect smoking inequalities among adolescents. A systematic review that assessed the equity impact of interventions/policies in youth smoking has concluded that increased tobacco price was the most effective in reducing socioeconomic inequalities in youth smoking, indicating that low SES youth were more responsive to tobacco price increase than high SES youth [32]. Likewise, in this study, it seems that the tobacco price increase might contribute to reduced smoking among Japanese adolescents from low familial SES groups, which resulted in the reduction of socioeconomic inequalities in smoking over time. During the study period, the tobacco price in Japan increased in 2010, 2014, and 2016. The price of the most popular cigarette brand in Japan, Mevius (the former name was Mild Seven before 2013) [33], increased from 300 yen in 2008 to 440 yen in 2016 (a 47% increase). A study among Japanese adults showed that the tobacco price increase in 2010 had a significant impact on smoking cessation across all socioeconomic subgroups and that this might be due to the affordable tobacco price for adults even after the price increase [33]. Yet, the increased tobacco price might have a greater impact on restricting tobacco purchase among Japanese adolescents who have less spending money, specifically the low familial SES groups.

Other tobacco control interventions/policies implemented during the study period were the installation of age verification vending machines using IC cards in 2008 [34] and the application of revised reference materials for school-based smoking, drinking, and drug abuse prevention instructions from 2010 to 2012 [35–37]. A Japanese study showed that age verification cards could not completely prevent adolescents from accessing tobacco products because adolescent smokers borrowed the IC cards from someone to buy cigarettes through vending machines [34]. Although school-based smoking prevention programs contribute to reduce smoking initiation among adolescents [38], a review showed that school-based interventions had mixed equity results [32]. In this study, it is not clear whether these two interventions contribute to reduce socioeconomic inequalities in smoking among Japanese adolescents.

In contrast to the familial SES indicators, time trends in absolute inequalities for school type persisted among boys and girls while relative inequalities increased over time. Although smoking prevalence decreased in both general and vocational schools, the absolute differences between both school types remained unchanged throughout the study period. It is noted that if the prevalence of the health outcome declines, relative differences may increase while absolute differences remain constant [39]. This pattern was demonstrated for school type in this study. The present findings were partly consistent with findings from previous studies that found no changes or increases in relative smoking inequalities for school type over time [12, 13, 20]. This may suggest that the different trends in smoking between the familial SES indicators and school type could be attributable to differential effects of different dimensions of SES; that is, the familial SES indicators reflect the social class of origin and school type represents the student’s individual social position [13, 20]. To reduce smoking inequalities for school type, smoking prevention policies and interventions should focus on vocational high school students.

To our knowledge, this study is the first to examine time trends in socioeconomic inequalities in smoking among Japanese adolescents. Nonetheless, several limitations should be noted. First, we assessed adolescent smoking using a self-reported single question. Although the question’s reliability has been confirmed, it is uncertain whether the question most successfully measured current smoking. This point might bring misclassification or underestimation of adolescent smoking in this study. In addition, we did not ask if students had used new tobacco-related products, such as electronic cigarettes (e-cigarettes) and heat-not-burn tobacco products. Although cigarette smoking among adolescents has declined, e-cigarette use among adolescents has been increasing [40]. A recent study in Japan also pointed out that e-cigarette use is gaining popularity among Japanese adolescents and many adolescents use new tobacco-related products only [41]. Thus, the overall declining trends in smoking may suggest that new tobacco-related products might be replacing cigarette smoking [42]. Second, we could not assess household income as an SES indicator. Thus, time trends in smoking inequalities for direct monetary aspects of SES are unknown. Third, we could not examine which father’s or mother’s education level was more important to adolescent smoking. Previous studies suggested that father’s education, but not mother’s education was related to adolescent smoking [43] and father’s education might be a stronger familial SES indicator than mother’s education was [44]. The present study showed that more than 70% of parental education included father’s education and the proportions were stable over time (data not shown). Thus, our indicator of parental education may partially reflect the role of the familial SES in adolescent smoking. Finally, since the study participants were exclusively from public high schools in Okinawa Prefecture, which is one of the poorest prefectures in Japan, the generalizability of the present findings to adolescents in Japan as a whole may be limited. As a cross-national study reported that smoking inequalities were much larger in the lowest income countries [45], smoking inequalities in Okinawa, which presents the worst SES indices, such as prefectural income per capita, unemployment rate, and the proportion of senior high graduates going to further education [46], may be prominently manifested. Meanwhile, it is unknown how the extent of poverty may have affected the trends in smoking inequalities. At any rate, our findings obtained from the poorest area may be unique and valuable for understanding and tackling socioeconomic inequalities in adolescent smoking.

## Conclusions

Smoking prevalence among Japanese adolescents has been declining and the absolute socioeconomic inequalities in smoking for parental education and family structure decreased between 2008 and 2016. In contrast, the relative socioeconomic inequalities for school type increased over time. Our findings suggest that time trends in socioeconomic inequalities in smoking varied by absolute and relative measures and that further policies and/or interventions to reduce smoking inequalities should focus on the context of schools, especially in vocational high schools.

## Data Availability

The dataset used during the current study is available from the corresponding author on reasonable request.

## Abbreviations

SES: Socioeconomic status
CI: Confidence interval
PD: Prevalence difference
PR: Prevalence ratio
SII: Slope index of inequality
RII: Relative index of inequality
